# Sensing-Applications of Surface-Based Single Vesicle Arrays

**DOI:** 10.3390/s101211352

**Published:** 2010-12-13

**Authors:** Sune M. Christensen, Dimitrios G. Stamou

**Affiliations:** 1 Bio-Nanotechnology Laboratory, Department of Neuroscience and Pharmacology, University of Copenhagen, 2100 Copenhagen, Denmark; 2 Nano-Science Center, University of Copenhagen, 2100 Copenhagen, Denmark; 3 Lundbeck Foundation Center for Biomembranes in Nanomedicine, Department of Neuroscience and Pharmacology, University of Copenhagen, 2100 Copenhagen, Denmark; 4 Center for Pharmaceutical Nanotechnology and Nanotoxicology, University of Copenhagen, 2100 Copenhagen, Denmark

**Keywords:** vesicles, single vesicles, model membrane systems, nanoreactors

## Abstract

A single lipid vesicle can be regarded as an autonomous ultra-miniaturised 3D biomimetic “scaffold” (Ø ≥ 13 nm) ideally suited for reconstitution and interrogation of biochemical processes. The enclosing lipid bilayer membrane of a vesicle can be applied for studying binding (protein/lipid or receptor/ligand interactions) or transmembrane events (membrane permeability or ion channel activation) while the aqueous vesicle lumen can be used for confining few or single macromolecules and probe, e.g., protein folding, catalytic pathways of enzymes or more complex biochemical reactions, such as signal transduction cascades. Immobilisation (arraying) of single vesicles on a solid support is an extremely useful technique that allows detailed characterisation of vesicle preparations using surface sensitive techniques, in particular fluorescence microscopy. Surface-based single vesicle arrays allow a plethora of prototypic sensing applications in a high throughput format with high spatial and high temporal resolution. In this review we present a series of applications of single vesicle arrays for screening/sensing of: membrane curvature dependent protein-lipid interactions, bilayer tension, reactions triggered in the vesicle lumen, the activity of transmembrane protein channels and biological membrane fusion reactions.

## 1. Introduction

**Figure 1.**
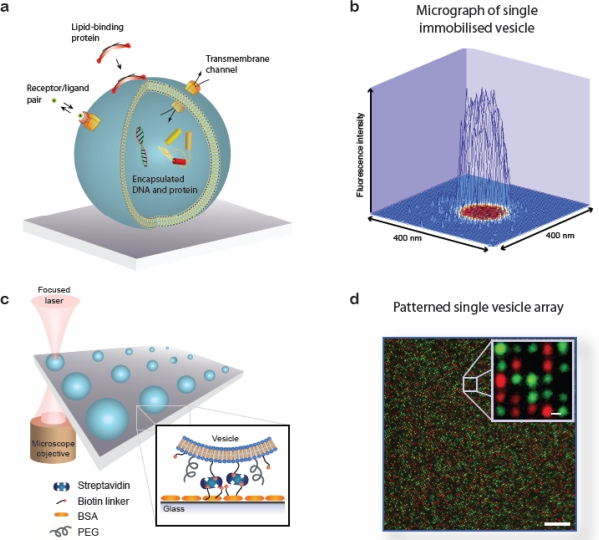
Surface-based lipid vesicle systems. **(a)** Sketch showing examples on various biochemical processes reconstituted in an immobilised vesicle. **(b)** Surface plot of the fluorescence intensity of a vesicle stained with a membrane anchored dye. Quantification of the fluorescence signal of single vesicles provides access to information on vesicle size and allows following reactions taking place at the membrane or within the lumen. **(c)** Single vesicle array interrogated by fluorescence microscopy. **(d)** Fluorescence micrograph of a single vesicle array functionalised with two populations of vesicles (red and green labels, respectively) [1]. Bars: 10 μm and 0.5 μm. d was adapted from reference [1] (Copyright Wiley-VCH Verlag GmbH & Co. KGaA. Reproduced with permission).

A lipid vesicle is comprised by an aqueous lumen confined by a lipid bilayer envelope. The close similarity to the overall architecture of cells makes (synthetic) vesicles an ideal platform for the reconstitution of biochemical processes in a native-like and well-defined model membrane system. A vesicle can be readily derivatised with organic and inorganic moieties thus creating an autonomous complex system of nanoscopic dimensions. This biomimetic “scaffold” can host motifs that permit surface immobilisation of the vesicle, identity encoding, monitoring of biorecognition processes taking place at or within the bilayer, probing of transmembrane transport and recording of biochemical reactions in general, see Figure 1(a). The possibility to simultaneously integrate and miniaturise at the nanometre scale allows one to perform a broad variety of experiments using femto- to zeptolitre (10^−15^L–10^−21^L) sized vesicle nanocontainers. Arraying of single vesicles on solid supports [Figures 1(b–d)] permits examination of individual vesicles in a high throughput format using fluorescence microscopy, thus harvesting the full potential of vesicles for ultra-miniaturisation of biochemical assays [1].

Extensive reviews on lipid vesicle production [2], characterisation [3] and the fabrication of surface-based vesicle systems [4] can be found elsewhere. In addition, excellent reviews have been published on other types of soft matter based nanocontainer systems including polyelectrolyte capsules [5], block-copolymer vesicles [6] and macromolecular boxes [7]. Here we focus on applications of surface-based arrays of single lipid vesicles. The review is structured as a series of examples on screening/sensing of: curvature dependent protein-lipid interactions, bilayer tension, reactions triggered in the vesicle lumen, the activity of transmembrane protein channels and biological membrane fusion reactions.

## 2. Sensing-Applications of Single Vesicle Arrays

### 2.1. Screening of Lipid-Protein Interactions as a Function of Membrane Curvature

Recently, it has become clear that membrane curvature has dramatic effects on a variety of membrane associated biological processes, e.g., enzymatic activity [8,9], membrane fusion [10] and lipid-protein interactions [11–13]. Traditionally, these phenomena have been investigated by measuring the average behaviour of reconstituted proteins in the presence of vesicles extruded through polycarbonate membranes of different pore diameters. We have, however, shown that such preparations in general have on the order of 70% overlapping size distributions [14] which seriously bias the result of any ensemble assay for screening of membrane curvature dependent effects.

To solve this problem we developed a fluorescence microscopy based calibration scheme for obtaining the size of single immobilised vesicles via the fluorescence intensity of a dye in the vesicle membrane (vesicle diameter ∝√ membrane area ∝√fluorescence intensity) [14,15]. From this procedure we established a single vesicle assay for screening of membrane curvature dependent protein-lipid binding reactions; in this assay each single vesicle immobilised at a surface provides a data point for a unique membrane curvature and a spectrum of curvatures can be screened in one experiment [16,17]. Of critical importance for this application we demonstrated that vesicles are intact [18] and non-deformed [19] upon immobilisation under appropriate conditions. The hallmark of the single vesicle assay is that it provides information on heterogeneous behaviour among individual vesicles that is averaged out in conventional ensemble measurements [12,13].

**Figure 2.**
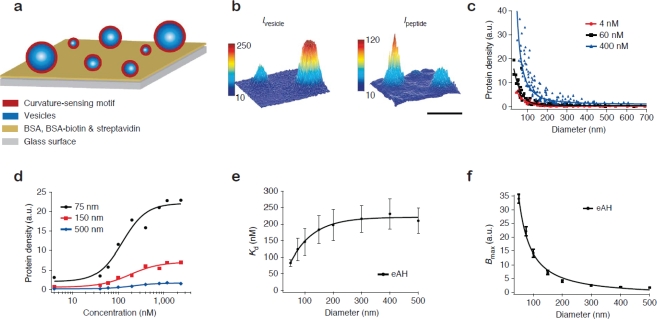
Screening of membrane curvature dependent protein-lipid interactions. **(a)** Vesicles of different size, and therefore different membrane curvature, were immobilised on a glass coverslip and incubated with a curvature-sensing motif. **(b)** Single vesicle positions and sizes (curvatures) were assessed from the fluorescence intensity of a lipophilic dye incorporated in the membrane (left). The amount of bound fluorescently labelled protein (in the shown example the amphipathic helix containing N-terminal domain of endophilin A1, eAH) was recorded in a second channel. The surface plots of intensity clearly demonstrates curvature specific binding, *i.e.*, the small vesicle to the left accommodated much higher density of protein than the large vesicle to the right. Bar: 1 μm. **(c)** Density of bound protein as a function of vesicle diameter for three different protein concentrations. **(d)** Binding curves as a function of vesicle diameter. **(e,f)** *K_d_* and *B_max_* as a function of vesicle diameter extracted by fitting of binding curves as illustrated in (d). We acknowledge Nature Chemical Biology where the material in this figure originally appeared [17].

Figure 2(a) shows the format of the single vesicle assay [16,17]. Vesicles of different size, and consequently different membrane curvature, are immobilised on a solid support and incubated with the protein of interest. Single vesicles are localised from a fluorescent label in the membrane [Figure 2(b), left]. With the aid of the aforementioned size calibration scheme [14] quantitative information on vesicle diameters is assessed from the intensity of the membrane label. The amount of dye-labelled protein bound to each vesicle is recorded in a second channel [Figure 2(b), right]. The ratio of the integrated intensity of the protein and the vesicle signal reveals the density of protein bound on each single vesicle and by collecting these data for all vesicles at the surface a membrane curvature sensing graph can be constructed. Figure 2(c) shows vesicle binding data for the amphipathic helix containing N-terminal domain of endophilin A1 (eAH) which is a well-documented sensor of membrane curvature [17].

The single vesicle platform permits the unambiguous evaluation of binding curves for different membrane curvatures [Figure 2(d)]. By fitting the binding curves Hatzakis *et al.* [17] quantified the curvature dependence of the apparent dissociation constant (*K_d_*) and the *B_max_* for binding of eAH to vesicles [Figures 2 (e,f)]. A prominent conclusion from this study was that the gain in free energy upon binding to a highly curved membrane is on the order of thermal fluctuations thus challenging the traditional view that membrane curvature sensing by amphipathic helixes is driven by affinity [20,21]. Surprisingly, the single vesicle results show that the increased density of protein on highly curved vesicles is in fact due to an increase in the saturation density (*B_max_*). Similar conclusions were reached for proteins that bind the membrane through lipidated residues [17]. Based on these results it was proposed that small vesicles have an increased number of binding sites due to the higher probability of forming a defect on a highly curved (highly tensed) bilayer as compared to a flat membrane.

A rather surprising observation from screening of protein binding on single vesicles was that for given conditions not all vesicles in the assay admitted protein [16]. The absolute number of vesicles that admitted protein was found to depend on vesicle size and the applied protein concentration [16]. This result has broad implications for ensemble assays in which such fractional binding behaviour would greatly obscure the interpretation of the average density of bound protein. For an in-depth survey of the single vesicle membrane curvature screening assay see references [12,13].

### 2.2. Immobilised Vesicles as Nanoscopic Sensors of Membrane Tension

**Figure 3.**
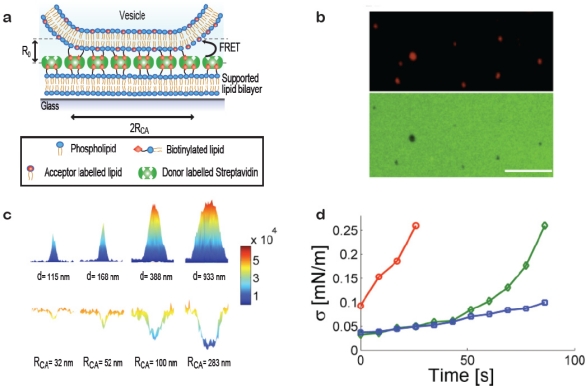
Measuring membrane tension on single vesicles by quantification of nanoscale contact areas formed between vesicles and a surface. **(a)** Sketch of the contact area formed between a vesicle and a streptavidin decorated supported bilayer. Quantitative information on the radius of the contact area (R_CA_) was obtained by measuring FRET between donor dyes on the streptavidin layer and acceptor dyes in the vesicle membrane. R_0_ depicts the Förster radius. **(b)** Fluorescence micrographs of vesicles with radius ≈100 nm (top) and the bilayer (bottom). FRET footprints are observed as a reduction in donor intensity. Bar: 5 μm. **(c)** Surface plots of fluorescence intensity of immobilised vesicles (top) and corresponding plots of fluorescence from the donor labelled streptavidin layer (bottom). By comparing theoretical and experimental results the degree of vesicle deformation upon immobilisation was evaluated. The obtained vesicle diameters, d, and the radii of the mapped contact areas (R_CA_) are indicated below the single vesicle intensity plots. **(d)** Time-resolved measurements of single vesicle contact areas used to evaluate the tension in the bilayer as a function of time. In this example tension increased due to strong laser illumination [22]. The graph shows data for 3 vesicles of different size (circles; vesicle diameter d = 362 nm, diamonds; d = 778 nm and squares; d = 1,272 nm). Figure adapted from [19].

When a vesicle attaches to a substrate it will deform provided the adhesive force is strong enough to bend the membrane. Deformation can be induced, e.g., by immobilising vesicles on substrates with high densities of streptavidin, Figure 3(a) (in the membrane curvature screening assay presented above low densities of streptavidin were used to ensure the absence of deformation). Our group has developed a method for quantifying subresolution contact areas between deformed vesicles and surfaces using Förster Resonance Energy Transfer (FRET) as a nanoscale ruler [19]. Knowledge of vesicle size and the area of the contact zone is sufficient to calculate the effective contact angle between the vesicle and the surface and from these parameters the tension in the bilayer can be readily obtained. Single immobilised vesicles can thus serve as nanoscopic sensors of membrane tension which provides a tool for probing mechanical changes in the membrane induced, e.g., by binding of proteins on the bilayer or thermotropic phase transitions.

Figure 3(a) shows a sketch of a vesicle deformed at a streptavidin decorated supported bilayer. Via a FRET pair in the streptavidin layer (FRET donor) and the vesicle membrane (FRET acceptor) with a Förster Radius (R_0_) matching the interbilayer separation quantitative information on the geometry of the contact zone was assessed. This was achieved by observing the reduction in donor intensity due to FRET [19]. Figure 3(b) shows a fluorescence micrograph of immobilised vesicles (top) and the streptavidin layer (bottom) revealing clear FRET footprints in the donor channel. A calibration measurement on giant vesicles adsorbed to the streptavidin layer yielded the FRET per contact zone area which, together with theoretical calculations of the expected FRET exhibited by a non-deformed vesicle, allowed mapping the radius of the contact zone for vesicles with sizes smaller than the optical diffraction limit [Figure 3(c)].

The method was used to probe the increase in bilayer tension upon exposing the vesicles to strong laser illumination which is known to increase the tension in dye containing bilayers [Figure 3(d)] [22]. As a function of time (exposure) bilayer tensions were observed to increase, providing a proof of concept for applying single immobilised vesicles as dynamic sensors of membrane tension. We anticipate that this method will prove particularly useful in establishing quantitative relations between biological processes occurring at or within the membrane and membrane tension.

### 2.3. Sensing/Screening Applications of Surface-Based Vesicle Reactor Systems

Recent years have seen a growing drive for the miniaturisation of volume based experiments. This drive is fuelled by the need of various biotechnological branches to reduce sample consumption and parallelise [23–26], investigate reactions in confined environments [27] and the rapidly growing field of single molecule biophysics [28]. Self-assembled lipid vesicles could provide viable fluidic solutions especially in niche areas where biocompatibility, miniaturisation and/or cost are critical. With dimensions ranging from the femto to the zeptolitre scale, approaching the globular size of proteins, vesicles may be considered the final frontier in miniaturisation of biochemical analysis. Lipid bounded compartments constitute the very fundament for life as we know it but the application of soft matter based compartments for the design of synthetic fluidic devices remains a field in its infancy. Below we will highlight a few examples that take advantage of surface-tethered vesicles as autonomous experimental volume systems for monitoring of confined enzymatic reactions, probing of transmembrane protein channels and single molecule experiments.

#### 2.3.1. Mixing Solutes inside Vesicle Reactors

To carry out controlled (bio)chemical reactions in vesicles it is necessary to master appropriate techniques for triggering the mixing of reactants. This problem has been solved by different strategies relying on electrical, mechanical, optical or thermal interaction with the lipid bilayer.

Early on Chiu *et al.* demonstrated how to mix the interior volume of selected single giant vesicle pairs (1–5 μm in diameter). Experiments were conducted in solution by trapping two vesicles using optical tweezers, bringing the vesicles together and inducing mixing of their initially separated content by delivering a voltage pulse and thereby causing the vesicles to fuse [29]. Orwar *et al.* used micromanipulators to establish fluid connections in the form of hollow lipid nanotubes (tube diameter ≈100 nm) between adjacent surface tethered giant vesicles [30]. Both mixing strategies rely on the use of micromanipulators (e.g., micropipettes or tweezers) to assemble and manipulate vesicles with diameters larger than one micrometre (volumes in the femtolitre regime). While micromanipulation is useful for precise manipulation of single vesicles it is by nature incompatible with parallelisation.

**Figure 4.**
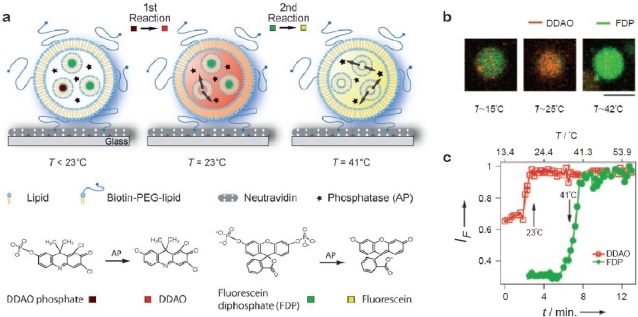
Self-assembled nanoscale fluidic device; consecutive enzymatic reactions triggered in single giant unilamellar vesicles. **(a)** A giant vesicle immobilised on a neutravidin-coated glass coverslip. Alkaline phosphatase (stars) was incorporated in the giant vesicle together with two different sets of small unilamellar vesicles (SUVs) each loaded with a different non-fluorescent substrate for the enzyme. One set of SUVs (T_t_ ≈ 23 °C) contained dichlorodimethylacridinone (DDAO) phosphate (dark red) and the second SUV population (T_t_ ≈ 41 °C) carried fluorescein diphosphate (FDP, dark green). An increase of temperature triggered the release of the substrates in two consecutive steps at the corresponding phase transition temperatures. After release from the SUVs, the substrates were confined in the lumen of the giant vesicle where they were processed to their respective fluorescent products by the enzyme. **(b)** Fluorescence micrographs of the process depicted in a. Bar: 10 μm. **(c)** Fluorescence intensity traces demonstrating sequential release of substrates and their conversion to fluorescent products in a single giant vesicle during a temperature ramp. Figure adapted from [32] (Copyright Wiley-VCH Verlag GmbH & Co. KGaA. Reproduced with permission).

Bolinger *et al.* developed a technique to release cargo from small unilamellar vesicles (SUVs) trapped inside the lumen of surface-tethered giant vesicles by taking advantage of the fact that bilayers become highly permeable at their phase transition temperature [31]. By relying on temperature as the trigger this strategy is well suited for addressing arrayed containers in parallel. Cargo was released from SUVs (volume ≈ 10^−18^ L) trapped in the lumen of single giant vesicles by elevating the temperature across the membrane phase transition of the enclosed SUVs (and not the giant vesicles). The nested system was fabricated simply by rehydration of the giant vesicle lipid mixture in a solution of the cargo-loaded SUVs. In a subsequent contribution it was shown that this platform can be used to titrate an enzyme trapped in the lumen of individual giant vesicles with different substrates in two consecutive steps by employing SUVs with different phase transition temperatures [32] [Figure 4(a)]. Figure 4(b) shows fluorescence micrographs of the process and Figure 4(c) shows the corresponding fluorescence traces. The possibility to use nested containers comprising SUVs with different lipid compositions allows one to release reactants in a programmed manner and perform a number of confined sequential reactions within single giant vesicles. This is the most advanced example to date on a self-assembled soft matter based nanoscale device.

To further tune the permeability properties of vesicles one could apply the plethora of membrane proteins and peptides naturally optimised for this purpose, e.g., channels or transporters with high selectivity to the size and polarity of molecules and ions [33]. Several examples on this topic can be found in the literature including: the peptide gramicidin A, used to control the pH inside immobilised vesicles [1], pore forming bacterial proteins (OmpF or alpha-hemolysin) used to continuously fuel a reaction taking place in the vesicle lumen [34–36] and reconstitution of the acceptor for λ-phage that allowed specific loading of DNA in targeted vesicles [37].

#### 2.3.2. Monitoring Activity of Transmembrane Protein Channels

Transmembrane protein channels that mediate the transfer of solutes over cellular membranes are of critical importance for uptake and secretion of material by cells and for signal transduction over the membrane. Robust assays for probing the activity of transmembrane channels are important for characterising their molecular properties and as tools in drug discovery. Single vesicle arrays could potentially provide access to functional screening of single transporters and ion channels in a massively parallel format. As a first step towards this vision Stamou *et al.* measured the activity of a pore forming peptide (Gramicidin A) on single immobilised vesicles [1]. Kuyper *et al.* in a subsequent contribution resolved the fine structure of passive proton permeation through the bilayer of single vesicles [38].

Adding an extra layer of complexity Pick *et al.* demonstrated activation of a ligand-gated ion channel (serotonin-gated 5-hydroxytryptamine type-3 receptor, 5-HT 3R) using “native” vesicles produced directly from the plasma membrane of cells overexpressing the receptor [39]. The vesicles were derived by incubating surface-attached cells with cytochalasin B (a vesicle budding promoter) during vortexing. Upon budding from the cells intact micrometre sized vesicles carrying multiple copies of the receptor attached to the substrate harbouring the cells. Addition of agonist triggered calcium influx, which was resolved by recording the fluorescence of an encapsulated Ca^2+^ indicator. A benefit of this approach is that it bypasses the need for protein purification although this is achieved on the expense of a well-defined lipid and protein composition.

Due to their nanoscopic dimensions it is generally challenging to directly resolve the kinetics of solute influx/efflux through transmembrane protein channels on individual SUVs (Ø ≤ 200 nm) due to the speed with which solutes are loaded/emptied once diffusive contact is established between the vesicle lumen and the surrounding medium. This property is, however, extremely useful for probing of channels with low permeability. Bränden *et al.* [40] in an elegant piece of work demonstrated quantitative measurements of the permeability properties of an aquaglyceroporin using evanescent-wave sensing. This was accomplished by immobilising a layer of vesicles carrying the reconstituted porin on a SPR (surface-plasmon resonance) compatible surface, which permitted label-free probing of the efflux kinetics of sugar alcohols into the immobilised vesicles. Because the vesicles in this setup were tethered at the sensor surface permeability of the porin towards several sugar alcohols could be measured on the same set of vesicles simply by exchanging the solution in the measurement cell.

With further improvement in sensitivity, e.g., by employing fluorescence microscopy based detection, it should in principle be possible to realise quantitative measurements on single channels in single SUVs. This would open a vista to a completely new platform for single channel recordings that would offer access to single channel analysis of slow transporters and dramatically increased data throughput (hundreds to thousands of vesicles measured in one experiment compared to a single data point per run in a conventional ensemble [41] or patch pipette setup [42]).

#### 2.3.3. Screening Single Molecule Reactions in Vesicles

Encapsulation of few or single water soluble biomolecules in surface-tethered vesicles accomplishes spatial fixation at a surface while retaining the molecules of interest in a native-like and well-defined environment (in contrast to direct surface conjugation which remains the prevailed method for single molecule experiments) [28,43]. Due to the spatial localisation the encapsulated entities can be followed over time, e.g., using vibrational [44] or fluorescence spectroscopy [43]. Boukobza *et al.* pioneered the use of vesicle encapsulation for single molecule studies by monitoring rotational dynamics of single encapsulated proteins [43]. Comparison of surface-conjugated and vesicle-encapsulated proteins revealed that at the surface the rotational freedom of the protein was greatly impeded while inside vesicles the protein exhibited rotational properties identical, within detection uncertainty, to that in free solution. Later on Rhoades *et al.* resolved structural fluctuations of single proteins trapped inside surface-tethered vesicles using single molecule FRET [45]. Vesicle encapsulation is now becoming an increasingly popular platform for single molecule experimenters, see references [46–48].

**Figure 5.**
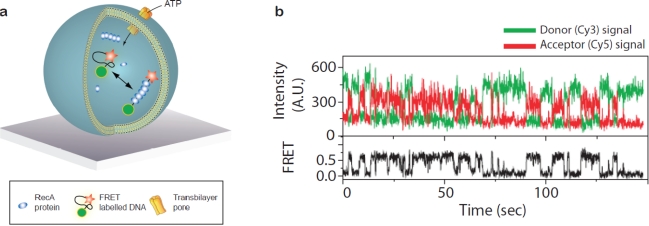
Single molecule recording of protein-DNA interactions driven by ATP inside a single vesicle. **(a)** A single strand of DNA labelled with a FRET pair encapsulated inside a surface-tethered vesicle together with the ATPase RecA (approximately seven copies per vesicle). In the cell RecA is active in DNA repair upon ATP regulated polymerisation on DNA. Upon RecA assembly the DNA was stretched giving rise to a reduction in FRET. Vesicles were made permeable to small molecules (<1 nm diameter) via pores in the membrane introduced either by utilising a lipid mixture with a phase transition at the temperature of the experiment [49] or using he bacterial toxin alpha-hemolysin [50]. Repeated polymerisation/dissociation reactions were triggered by exchange of ATP through the pores. **(b)** Fluctuations between stretched (low FRET) and unstretched (high FRET) states of the DNA. Figure b adapted from [49], copyright 2007 National Academy of Sciences, USA.

Taking advantage of the vesicle as a container for grouping reactants together Ha and co-workers used single vesicle encapsulation to characterise intermolecular association/dissociation between a single DNA strand and a handful of RecA proteins [Figure 5(a)] [49,50]. For the purpose of probing intermolecular reactions vesicle encapsulation is particularly useful in analysing low affinity interactions since even a single molecule in a 100 nm vesicle has a concentration of roughly 3 μM. By attaching FRET probes at the distal ends of the DNA strand folding and unfolding induced upon polymerisation of the RecA along the DNA was traced in real time [Figure 5(b)]. The reaction was fuelled by exchange of ATP/ADP over the vesicle membrane while retaining the proteins and DNA inside by rendering the membrane semipermeable either by employing lipids with a phase transition at the temperature of the experiment [49] or by inserting alpha-hemolysin pores in the bilayer [50]. In this way continued association/dissociation of the RecA upon ATP binding and subsequent hydrolysis could be detected enabling quantification of folding/unfolding rates.

Because vesicle composition can be easily controlled vesicle encapsulation should be an ideal experimental scene for investigating systematically how the local nanoenvironment influences the behaviour of biomolecules. Furthermore, single vesicle arrays open for the possibility to quantitatively examine the effect of spatial confinement on biochemical reactions since vesicle sizes can be accurately determined (see Section 2.1).

### 2.4. Screening of Membrane Fusion Reactions

A new promising application of the single vesicle platform has emerged in the study of biological membrane fusion reactions as encountered, e.g., in the secretory pathway of eukaryotic cells [51], neurotransmission [52] and virus infection [53]. The merging of the membrane of a transport vesicle or virus particle with a cellular target membrane is acentral step in all these pathways. In cells, the highly conserved family of SNARE proteins (SNAP receptors with SNAP defined as soluble N-ethylmaleimide-sensitive factor (NSF) attachment protein) is considered the core of the membrane fusion machinery. In preparation for fusion cognate SNAREs on the membrane of the vesicle (vSNAREs) and the target membrane (tSNAREs) assemble into bundles that bridge the two membranes, force them into close proximity and thereby facilitate their fusion [51,52].

The conventional assay to investigate SNARE mediated membrane fusion *in vitro* is the fluorometry based FRET lipid mixing assay [54]. This assay is based on the reconstitution of v- and t-SNAREs together with auxiliary proteins in two vesicle populations simulating the target membrane (*i.e.*, intracellular organelle or the plasma membrane) and the transport vesicle, respectively. One of the vesicle populations is labelled with a lipophilic FRET pair and lipid mixing (indicative of membrane fusion) is monitored upon dequenching of FRET donor fluorescence caused by the dilution of the fluorophores over a larger membrane surface upon fusion of labelled and unlabelled vesicles. Despite its merits, the lipid mixing assay suffers from the major drawback that it cannot discriminate between the docking and the fusion of vesicles due to spatiotemporal averaging. This shortcoming can be circumvented using surface-tethered vesicles which allow following docking and fusion of individual vesicle pairs in real time by tracking single interaction events using fluorescence microscopy [55–59].

**Figure 6.**
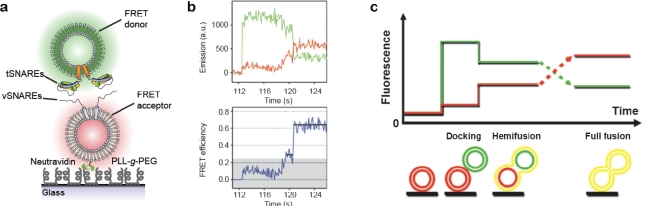
Single vesicle-vesicle fusion assay. **(a)** A population of vesicles carrying reconstituted vSNAREs is tethered to a solid support through biotin-neutravidin coupling and allowed to interact with a freely diffusing population of vesicles harbouring cognate tSNAREs. The surface is passivated with PLL-*g*-PEG to prevent non-specific interactions of the tSNARE vesicles and the surface. **(b)** Discrete steps of docking, hemifusion and full fusion as evidenced from the apparent FRET efficiency (bottom) calculated from donor (green) and acceptor (red) emission during fusion of a single vesicle pair. The abrupt increase in donor emission in the beginning of the trace marks vesicle docking. **(c)** Simplified sketch of the evolution of donor and acceptor emission and the configurations thought to be associated with each level of FRET. Figures (b,c) were adapted from [56], Copyright 2006 National Academy of Sciences, USA.

Figure 6(a) illustrates the setup in the single vesicle fusion assay as pioneered by Yoon, Ha and co-workers [56] (an alternative setup uses a supported membrane instead of immobilised vesicles, see references [60–62]). Vesicles functionalised with vSNAREs and labelled with a FRET acceptor dye are immobilised on a surface. The surface-tethered vesicles are then brought to interact with freely diffusing vesicles bearing cognate tSNAREs and a FRET donor dye. The relative degree of lipid mixing upon single vesicle interaction events is assessed by evaluating the apparent FRET efficiency [56]. With this assay Yoon *et al.* [56] succeeded in resolving intermediates of the fusion process as distinct levels of FRET [Figures 6(b,c)].

Single vesicle fusion assays have brought new insights on SNARE mediated fusion that previously were masked due to ensemble assaying such as the presence of fast (< 1 s) fusion events [56,57,60,63] and the importance of the membrane-integral domain of the neuronal Ca^2+^ sensor synaptotagmin-1 for achieving a fusion reaction sensitive to physiological levels of Ca^2+^ [58], as required for the release of synaptic vesicles at nerve terminals. Given its current success, it seems without doubt that single vesicle assays will become a standard in the field of reconstituted membrane fusion within the next five years.

## 3. Perspectives

A lipid vesicle comprises a 3D nanoscopic scaffold that can group together water soluble, hydrophobic or amphiphilic molecules. Their high biocompatibility makes lipid vesicles ideal model membrane systems for the reconstitution and characterisation of various biological processes. In addition to their potential as model membranes, the application of vesicles as ultra-miniaturised biochemical reactor systems has gathered attention from the single molecule community and from various biotechnological branches that could benefit from alternative technologies for reducing sample consumption and parallelise volume based experiments. Arraying of single vesicles at surfaces allows highly parallel examination of vesicle preparations by microscopy.

The ultimate vision of the experiments we have described in this review would be a high density single vesicle array (>10^8^ per cm^2^) of single nanoscopic multifunctional experimental units. The membrane of the vesicles would be used to monitor transmembrane events (e.g., ion channel activation) or binding of water soluble molecules (peripheral proteins, ligands, *etc.*). The lumen of the vesicles would be used to confine single macromolecules and observe folding, catalytic pathways of enzymes or more complex biochemical reactions. The reconstitution of signal transduction cascades as mediated, e.g., by G-protein coupled receptors (GPCRs) or the T cell receptor for antigen (TCR) comprises a particularly promising prospect that would be of significant importance both for gaining novel insight and for screening the activity of such reactions against libraries of drug candidates. Signal transduction is initiated at the membrane with the binding of a ligand to the extracellular part of the receptor and the signal is communicated downstream via interactions of the transmembrane receptor and intracellular components. The single vesicle array should be an ideal platform for reconstituting this type of reactions in an authentic synthetic environment and characterise them in a high throughput format with high sensitivity.

Immobilisation of intact and non-deformed vesicles has now become routine practise and methods to extract quantitative information from individual vesicles using fluorescence microscopy are in place. Furthermore, several solutions have been devised to access the vesicle interior and trigger solute mixing or allow exchange of reactants over the vesicles membrane.

The critical outstanding issue for the vision of fabricating a multifunctional biochip based on a high density vesicle array is encoding the identity of large numbers of different vesicles and thereby allow rapid multiplexing. The rise of second generation DNA sequencing platforms [23] has, however, made commercial technology available that would allow the massively parallel decoding of DNA tagged single vesicle libraries. Though challenges regarding production of vesicle libraries still remain to be solved the presented vision has now come within reach.
